# Is There a Rural Penalty in Language Acquisition? Evidence From Germany's Refugee Allocation Policy

**DOI:** 10.3389/fsoc.2022.841775

**Published:** 2022-06-02

**Authors:** Samir Khalil, Ulrich Kohler, Jasper Tjaden

**Affiliations:** ^1^Department Social Sciences, Faculty of Economics and Social Sciences, University of Potsdam, Potsdam, Germany; ^2^German Center for Integration and Migration Research (DeZIM), Berlin, Germany; ^3^Global Migration Data Analysis Centre, International Organization for Migration, Berlin, Germany

**Keywords:** refugees, allocation policies, rural, language acquisition, intergroup contacts, language courses, integration

## Abstract

Emerging evidence has highlighted the important role of local contexts for integration trajectories of asylum seekers and refugees. Germany's policy of randomly allocating asylum seekers across Germany may advantage some and disadvantage others in terms of opportunities for equal participation in society. This study explores the question whether asylum seekers that have been allocated to rural areas experience disadvantages in terms of language acquisition compared to those allocated to urban areas. We derive testable assumptions using a Directed Acyclic Graph (DAG) which are then tested using large-N survey data (IAB-BAMF-SOEP refugee survey). We find that living in a rural area has no negative total effect on language skills. Further the findings suggest that the “null effect” is the result of two processes which offset each other: while asylum seekers in rural areas have slightly lower access for formal, federally organized language courses, they have more regular exposure to German speakers.

## Introduction

Once asylum seekers arrive in Germany, they are distributed geographically across the German regions. The number of asylum seekers that each region receives is based on a quota system considering tax returns and population size in each region (Königstein key). The allocation of individuals across those defined regions occurs randomly. This policy is subject to much debate. The system resembles a lottery that may produce winners and losers. An emerging body of research suggests that the initial placement of asylum seekers shapes their further integration trajectories into society (Chiswick and Miller, 2002; Åslund and Rooth, 2007; Aksoy et al., 2020). Local contexts may vary substantially in terms of educational, labor market and social opportunities they provide for migrants (Edin et al., 2003; Beaman, 2012; Godøy, 2017; Martén et al., 2019; Braun and Dwenger, 2020). Several initiatives have been launched to assess the potential of taking additional characteristics into account when matching asylum seekers to localities with the aim to increase integration outcomes such as employment (Bansak et al., 2018).^1^ The societal benefits of improving geographic assignment appear large in light of the long-term disadvantage that asylum seekers and refugees face in terms of employment and earnings (Dustmann et al., 2017; Brücker et al., 2019; Brell et al., 2020).

In this study, we aim to explore the question whether asylum seekers that have been allocated to rural areas experience disadvantages compared to those allocated to urban areas. Some studies have shown that urban centers with a higher share of co-ethnic residents provide advantages in terms of economic integration (Martén et al., 2019). Higher concentration of co-ethnic networks reduce initial language barriers and information asymmetries when searching jobs. Urban areas may also provide more support to newcomers in terms of language learning opportunities or other support services in multiple languages. Rural areas–due to fewer available resources and fewer previous migration–may offer less support. Several initiatives have been launched in Germany to improve access to integration courses (providing language learning opportunities) in rural areas (Ohliger and Schweiger, 2019; Rösch et al., 2020; Fachkommission Integrationsfähigkeit, 2021). Research on co-ethnic networks and integration opportunity structures suggest that asylum seekers could be disadvantaged in rural areas. The available empirical evidence, however, is still limited (Rösch et al., 2020).

In this study, we explore potential rural penalties with a focus on language acquisition. Language skills are often highlighted as the main driver of positive integration trajectories (Esser, 2006; Kristen et al., 2016; Kosyakova et al., 2021) as they facilitate job searches, social integration, and correspondence with authorities or navigation of host-country institutions (Espenshade and Fu, 1997; Martinovic et al., 2009; Alba et al., 2011). In particular, we will assess several pathways that may explain differences in language acquisition between rural and urban locations based on a causal model illustrated by Directed Acyclic Graphs (DAG) (Elwert, 2013). We derive testable hypotheses based on language learning models initially developed by Chiswick and Miller (2001) and later extended and applied by various authors (e.g., Kristen et al., 2016; Kosyakova et al., 2021).

Based on large longitudinal survey data in Germany (SOEP IAB-BAMF refugee sample; *N* = 13,187), we first test whether there is, indeed, a rural penalty in language acquisition of asylum seekers. Second, we explore whether potential urban-rural disparities are related to differences in social networks (i.e., exposure to German speakers) and learning opportunities (access to language courses). Research has shown that contacts with natives (Bauer et al., 2005; Heath et al., 2008; Danzer and Yaman, 2013) and participation in language courses (Clausen et al., 2009; Vroome and van Tubergen, 2010; van Tubergen, 2010; Kaida, 2013; Hoehne and Michalowski, 2016; Sarvimäki and Hämäläinen, 2016; Auer, 2018; Lochmann et al., 2019; Arendt et al., 2020; Kosyakova and Brenzel, 2020) have strong and lasting effects on integration outcomes such as language acquisition and employment.

While previous research has largely discussed individual mechanisms in isolation, we propose a broader framework that incorporates different forms of opportunity structures for language acquisition of refugees depending on their geographic location. The geographic dimension of integration of refugees was neglected previously, largely due to the lack of suitable data sources. There is ongoing discussion on how particular contexts shape integration outcomes for example with respect to concentration of co-ethnic/ migrant networks, contacts to non-migrants, local employment rates, and state-funded integration support initiatives.

Our results show that (1) there is no overall rural penalty in refugees' language acquisition in Germany, (2) both contact with Germans and participation in different forms of language courses proof to be highly effective in increasing refugees' language acquisition and (3) intergroup contact with Germans is significantly more likely in rural areas while official course participation is somewhat less likely. Overall, it can be concluded that language learning in rural areas runs to a greater extent via contacts with Germans, while in urban areas institutional services are a more relevant factor.

In addition to advancing understanding of how local contexts shape integration of refugees, these results have implications for policy. The federal government is responsible for the allocation of refugees across regions and regional authorities are responsible for allocation of refugees to districts. The findings reject the claim that refugees are disadvantaged in rural areas in terms of language acquisition, partly because higher exposure to German speakers offsets marginally lower access to formal language courses. The results also suggest that further investment in courses in rural areas and more opportunities for interactions with Germans in urban areas could accelerate language acquisition of refugees and thus maximize integration benefits for refugees and society.

## Theory

According to the Chiswick-Miller language learning model, host-country language acquisition is a function of efficiency, incentives and exposure (Chiswick and Miller, 2001). *Efficiency* captures factors that facilitate individual language learning such as prior education attainment, young age, and cognitive skills. *Incentives* reflect the motivation of the language learner and are driven by expected economic (i.e., income) and non-economic (home-country attachment, social exclusion) returns. The incentive dimension incorporates costs associated with language learning such as fees for instruction, material costs or opportunity costs associated with delayed transition to gainful employment. Incentives are commonly modeled as a (rational) cost-benefit calculation by the individual migrant.

*Exposure –* the main dimension of interest for this study – refers to “the degree to which the new language is present in contexts that immigrants encounter” (Kosyakova et al., 2021). Exposure incorporates structural language learning opportunities such as courses and interactions with native-speakers.

In this study, we are interested in potential disadvantages of residing in a rural area with regards to language learning among recently arrived asylum seekers. In particular, we are interested how exposure to German native-speakers (through every-day interactions) and access to formal language courses mediate potential effects of location on language learning.

In the following, we put forward our theoretical arguments formalized by the means of Directed Acyclic Graphs (DAGs). DAGs are a tool to illustrate the causal model, make assumptions transparent and derive formal rules for selecting control variables (Elwert, 2013; Morgan and Winship, 2014).

**Figure 1:**
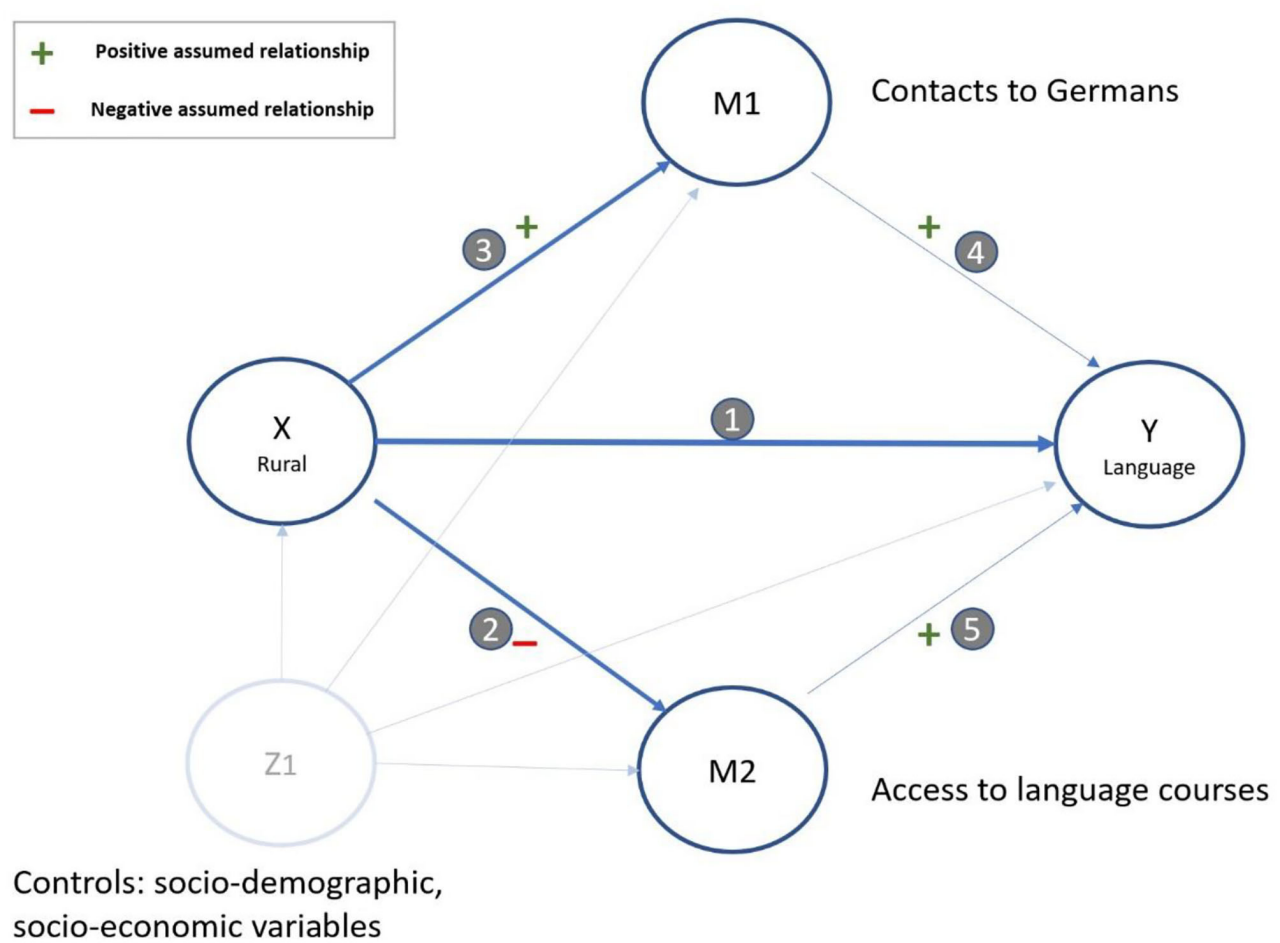
Directed Acyclic Graph of the proposed causal model.

The main interest of this study is the total causal effect of location (urban vs. rural) on language acquisition (in the form of skills) (path 1 in Figure 1). More explicitly, we are interested in the role of the two indirect effects (mediators) of language courses (M2) and contacts to Germans (M1). The positive effect of contacts with native speakers (path 4) and language course attendance (path 5) on language acquisition is already well established (Niehues et al., 2021; Kristen et al., 2022). The focus of this study is how refugees living in rural areas are affected by both courses and contacts relative to refugees living in urban areas in terms of language acquisition.

There are several reasons to assume a negative effect of a rural location on *language course participation* (path 2). First, rural communities often provide less assistance in language learning. Rural communities have fewer resources to fund language learning opportunities (Schader Stiftung, 2011; Ohliger and Schweiger, 2019; Scheible and Schneider, 2020). Second, even if resources are available, rural areas do not benefit from scaling effects due to lower population size and density. In other words, if fewer asylum seekers are present, certain investments in support measures such as integration courses may not be deemed cost-effective (Ohliger and Schweiger, 2019; Scheible and Schneider, 2020). Third, rural areas have lower levels of previous migration which indicated less experience with managing diversity and established support policies (Rösch et al., 2020). This could mean that available support is of lower quality or consistency. Fourth, courses may be available in neighboring localities but too difficult to access given the distance and lower public transport provision (Scheible and Schneider, 2020). Fifth, migrants in rural areas may be less incentivized to learn the languages because there are lower expected returns to the investment given that fewer and worse jobs are available in rural areas compared to urban areas.

In contrast, there are several reasons to believe that asylum seekers living in rural areas have *more exposure to native-speakers* (path 3) compared to asylum seekers living in urban settings. First, the opportunity to interact with other co-ethnics is likely smaller because the concentration of migrant groups is historically lower in rural areas compared to cities (Luft, 2011; Berlinghoff, 2018). Many migrant groups in Germany settled in cities following the economic boom after World War II. Still today the proportion of migrants is much higher in cities than in the countryside (Beauftragte der Bundesregierung für Migration, 2021). Living in urban areas may offer newcomers more employment opportunities and more inter-ethnic support in navigating host-country society, however, it may be a disadvantage in terms of language learning because of fewer interactions with native speakers (Chiswick and Miller, 1996, 2002; Bauer et al., 2005; Kanas et al., 2012; Danzer and Yaman, 2013; Chiswick and Wang, 2019). Public infrastructure in cities (in terms of mobility, basic services and health) reduces reliance on personal social contacts in general. In rural areas the principle of mutual assistance between neighbors, friends and families is more important in light of weaker public infrastructure. In rural areas, it may therefore seem more likely that asylum seekers will need to enter into contact with German speakers, e.g., with their neighbors, in order to help each other in everyday life. Finally, an opposing relationship between rurality and contact with Germans is also conceivable. It is known from previous studies that populations in cities tend to be more tolerant of migration (Bangel et al., 2017). This openness could also lead to more frequent and more intensive contact. It remains an empirical question for this study which of the opposing associations overweigh in this regard.

In sum, we suggest that contacts to Germans and access to formal integration courses condition any effect from rural location on language acquisition (full mediation). Based on these theoretical reflections, we consider the following potential effects of rural area on language acquisition:

- **Rural penalty**: Asylum seekers and refugees in rural areas are disadvantaged in terms of language acquisition because negative mediation outweighs positive mediation. According to theory, in this scenario, effects from reduced access to language courses cannot be sufficiently compensated by increased contact with Germans.
- **Rural premium**: Asylum seekers and refugees in rural areas are advantaged in terms of language acquisition because positive mediation outweighs negative mediation. According to theory, in this scenario, effects from reduced access to language courses are successfully overcompensated by increased contact with Germans.
- **Compensation effect:** Being assigned to a rural location has no overall effect on language acquisition because positive and negative mediation offset each other, i.e., reduced access to language courses is equally compensated by increased contact with Germans.

An obvious contention that may threaten the causal interpretation of our findings is selection. Asylum seekers with particular (observable or unobservable) characteristics may be more likely to live in rural areas (Rösch et al., 2020). The same characteristics may be associated with better access to integration courses and interactions with Germany (**Z1**). For example, younger and more educated migrants may sort themselves into urban contexts to seek better employment and more attractive lifestyle opportunities. Parents with children may prefer rural areas with lower living costs and cheaper rents while parents, particularly women, have less availability to participate in language courses (Tissot et al., 2019). Certain asylum seekers from countries with low recognition rates facing legal obstacles to enter formal language courses could be concentrated in certain locations in Germany.

To overcome this problem, we make use of a unique feature of the German asylum seeker distribution system (sometimes referred to as settlement policy in other countries). In Germany, asylum seekers are randomly allocated to a particular region and then quasi-randomly distributed further to counties. No information about the individual asylum seekers is considered when assigning a location. This is an ideal situation for causal identification resembling a natural experiment. In addition, according to a new policy, the residence of recent asylum seekers is limited to their place of first assignment. This mobility limitation assures that the composition of asylum seekers is similar across localities which – in theory – should also render the population assigned to rural and urban areas non-selective. For any potential imperfections of this random assignment that may occur in practice, we take several measures further described in the following sections. Furthermore, we conduct additional analyses in sub-samples in which we control for factors that reflect the intention and experience of living in the countryside, thus ruling out further potentially endogenous variation (Supplementary Figure A1).

## Data and Methods

### The IAB-BAMF-SOEP Refugee Survey

The IAB-BAMF-SOEP Refugee Survey is a longitudinal household survey of asylum seekers and refugees in Germany and was launched in 2016 (Brücker et al., 2017; Liebig et al., 2021). The target participants entered Germany between January 2013 and June 2019 and applied for asylum. The survey covers the respondent and all household members of the respondent. The survey aims to collect information on the living conditions of protection seekers in Germany. This includes among other things information on language acquisition, schooling and vocational training, psychological and social factors as well as participation in the labor market. For this study, the rich information regarding the use of language courses is particularly relevant, as well as the information regarding different forms of intergroup contacts with Germans. To ensure that a possible lack of German skills did not pose a hurdle in responding to the survey, respondents were offered a choice of six more language versions of the questionnaire (Arabic, Kurmanji, Farsi, Urdu, Pashto and English) (Brücker et al., 2017).

For our analyses, we use all available survey-years between 2016 and 2019. From originally 18,342 person-survey-years, we make use of 13,187 observations that contain our variables of interest. Overall, observations are nested within 6,985 individuals surveyed once or repeatedly between 2016 and 2019.

### Measurements

Our main dependent variable is language proficiency in German for which we use respondents' self-assessment: across three separate items, individuals are asked how well they can speak, write, and read in German, each on a 5-point scale from *not at all* to *very well* (SOEP Group, 2020). We use all three variables to create an additive index which we allow to vary between 0 and 1 with greater values indicating higher levels of language proficiency (for a similar approach, see Kosyakova et al., 2021). To test the robustness of this measure, we also re-estimate the main models using the interviewer's assessment of the respondents' language ability. Results are discussed in the following section and reported in the Supplementary Materials.

**Figure 2:**
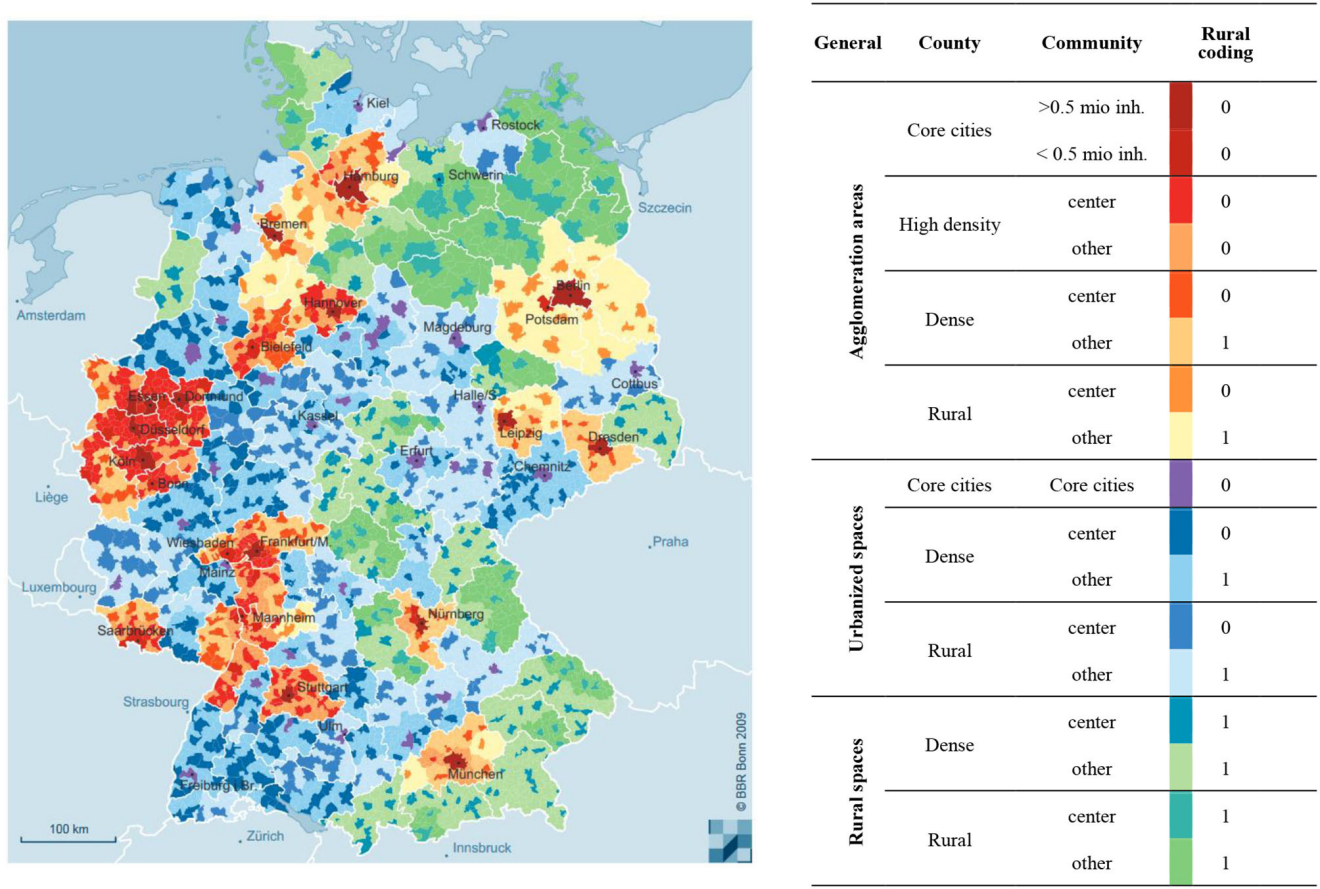
Regional classifications available in SOEP data. Displayed are the 17 residential-structural community types in Germany, introduced by the Federal Institute for Research on Building, Urban Affairs and Spatial Development and available within scientific-use files for SOEP-surveys with regards to their place of residence. Figure adopted from BBR (2009), rural coding based on own consideration.

Our central independent variable captures whether refugees live in *rural areas* at the time of their interview. A typification of rural areas can vary greatly depending on the underlying social, economic and spatial indicators (Küpper, 2020). For this study, it is especially important to distinguish local contexts according to their population density, resources, access to public transportation, provision of language courses and the concentration of inter-ethnic communities that arrived previously. Therefore, we use a typification of the Federal Institute for Research on Building, Urban Affairs and Spatial Development, exhausting variation across the more than 10,000 municipalities in Germany, providing a very fine resolution. Municipalities are classified in a nested manner within counties (core cities, dense counties and rural counties) and more general regional types (agglomeration areas, urbanized spaces and rural spaces), see Figure 2. While the two highest levels are classified primarily based on population density, there is a differentiation implemented on the lowest level of municipalities, indicating whether a given municipality represents a so-called regional center or not (*Oberzentrum* or *Mittelzentrum*). Regional centers have a supraregional significance and are usually characterized by a higher level of facilities in various areas, such as culture and education, health, transport connections or administration and authorities (Einig, 2015). In principle, we define rurality when refugees do *not* live in such regional centers. We partly deviate from this definition in highly dense agglomeration areas, since a good accessibility of centers can be assumed, i.e., refugees in these areas likely benefit from the nearby centers and the social infrastructure available to them (e.g., the public transportation supply). Likewise, we define centers in very peripheral areas as rural, since there likely is no equivalent supply of social facilities and infrastructure present as compared to urban areas.

The two main mediating variables in our model are “contact to Germans” and “participation in language courses.” We measure contact with Germans based on the SOEP's question on *how often respondents spend time with German people* (SOEP Group, 2020). The original 6-point-scale runs from “never” to “every day.” We define a dummy variable indicating the top-2 values “several times per week” or “every day” and contrasting them to remaining options of “every week” or less often. To test the robustness of these measures, we also re-estimate the main models using alternative measures of contacts with friends, colleagues and neighbors (see section Further analyses & robustness checks).

Participation in language courses is measured based on several SOEP survey items regarding participation in various different types of courses. This includes the general official language course, organized by the Federal Office for Migration and Refugees^2^ (BAMF) as well as various specific centrally organized course formats, for instance targeted at young refugees, female refugees or with focus on occupational language development (SOEP Group, 2020). We include three separate variables to compare possible differences across regions: (1) we include a variable indicating *any course visit* irrespective of the specific form (2) we include a variable indicating the official (BAMF) course visit and (3) one variable indicating the report of “other” course visits which were not administered by the BAMF. The latter could therefore include locally organized efforts to promote the language acquisition of refugees and are therefore of particular importance.

As discussed above, the random spatial distribution of refugees may be imperfect in practice. Furthermore, even if randomly allocated, asylum seekers and refugees may sort themselves into courses or intergroup contact, depending on various individual characteristics. To isolate the effect pathways from such possible confounders, we therefore include a set of control variables. Thus, we include information on socio-demographic factors *sex, age* and *educational levels*, migration-specific factors *country of origin, years since immigration* and *legal status*, as well as indications on *partnerships, number of children* and *moves since the last survey*.

### Empirical Strategy

Each of the paths in the overall model (Figure 1) is estimated separately by respecting the backdoor criterion (Elwert, 2013). This is accompanied by assumptions on which factors should be controlled for depending on the path considered. For estimating the total causal effect from rural assignment on language acquisition, we control only for confounders (Z1) to consider any imperfections in the random allocation process that may occur. The same applies to path 2 (rural assignment → course visits) and path 3 (rural assignment → contacts to Germans) for which we only control for individual confounders (Z1) to eliminate sorting effects after geographic allocation. For path 4 (contact → language acquisition) and 5 (course visit → language acquisition), we additionally control for rural assignment (X), and we, respectively, control for course visits in path 4 and for contacts in path 5 to block pathways between the two. The implications of this modeling in the context of potential two-directional effects between contacts and courses are discussed in the section Further Analyses and Robustness Checks.

To estimate the treatment effects of all five hypothesized paths, we use Stata's effect command using the regression adjustment (RA) estimator (StataCorp, 2021). RA estimators implement a two-step approach in which separated regression models of the outcome on a set of covariates are fitted for each treatment level. Using the coefficients of these separated regressions, the predicted values of the outcomes are calculated, including the out-of-sample predictions for the observations with the other treatment level(s). Each set of predicted values are then considered as the potential outcome for the respective treatment level, and the difference in the sample means of a pair or potential outcomes are taken as estimate for the corresponding population average treatment effect (Cattaneo, 2010; StataCorp, 2021). In comparison to standard multiple regression, this approach does not assume homogeneous treatment effects across levels of covariates. To allow for intra-individual correlation of standard errors, they are clustered on respondent-level.

### Summary Statistics

Table 1 illustrates weighted summary statistics on most relevant variables included in later analyses based on our analytic sample comprising 13,187 observations. Summary statistics are differentiated by our main independent variable (i.e., whether refugees live in either rural or urban areas). Overall, most factors seem relatively balanced across regions which highlights the importance of administrative distribution measures, discussed in the introduction. Nevertheless, some minor differences can be noticed. Thus, refugees living in rural areas are younger (30.5 vs. 31.3, *p* < 0.05), less likely to be highly-educated (18.6 vs. 21.8%, *p* < 0.05) or from Syria (35.3 vs. 44.2%, *p* < 0.05).

**Table 1:** Summary statistics.

		**Urban areas**					**Rural areas**			
**Variable**	**Group**	**MEAN**	**SD**	**MIN**	**MAX**		**MEAN**	**SD**	**MIN**	**MAX**
Female		0.277	0.447	0	1		0.264	0.441	0	1
Age		31.290	10.687	17	97		30.494	9.690	17	79
In relationship		0.366	0.482	0	1		0.351	0.477	0	1
Years since Immigration		2.153	1.015	0	5		2.071	0.999	0	5
Number of children		1.167	1.766	0	15		1.144	1.742	0	19
Educational attainment	Low	0.387	0.487	0	1		0.411	0.492	0	1
	Middle	0.395	0.489	0	1		0.403	0.491	0	1
	High	0.218	0.413	0	1		0.186	0.390	0	1
Country of birth	Syria	0.442	0.497	0	1		0.353	0.478	0	1
	Iraque	0.089	0.284	0	1		0.089	0.284	0	1
	Afghan.	0.118	0.323	0	1		0.148	0.355	0	1
	Eritrea	0.048	0.213	0	1		0.054	0.226	0	1
	Other	0.303	0.460	0	1		0.357	0.479	0	1
German language skills		0.495	0.254	0	1		0.469	0.249	0	1
Course visit	Official	0.438	0.496	0	1		0.380	0.500	0	1
	Inofficial	0.312	0.496	0	1		0.342	0.486	0	1
	Any	0.691	0.463	0	1		0.673	0.474	0	1

*Displayed are weighted summary statistics for refugees living either in regional centers (n_prs−years_ = 9, 261) or in rural area (n_prs−years_ = 3, 926)*.

Regarding our main theoretical variables, there is no significant difference in German language skills between rural and urban locations. Frequent contact to Germans is significantly more likely in rural areas and at the same time, any language course participation and specifically official course participation is less likely for respondents in rural areas while unofficial course visits are slightly more likely.

## Results

### Main Models

**Figure 3:**
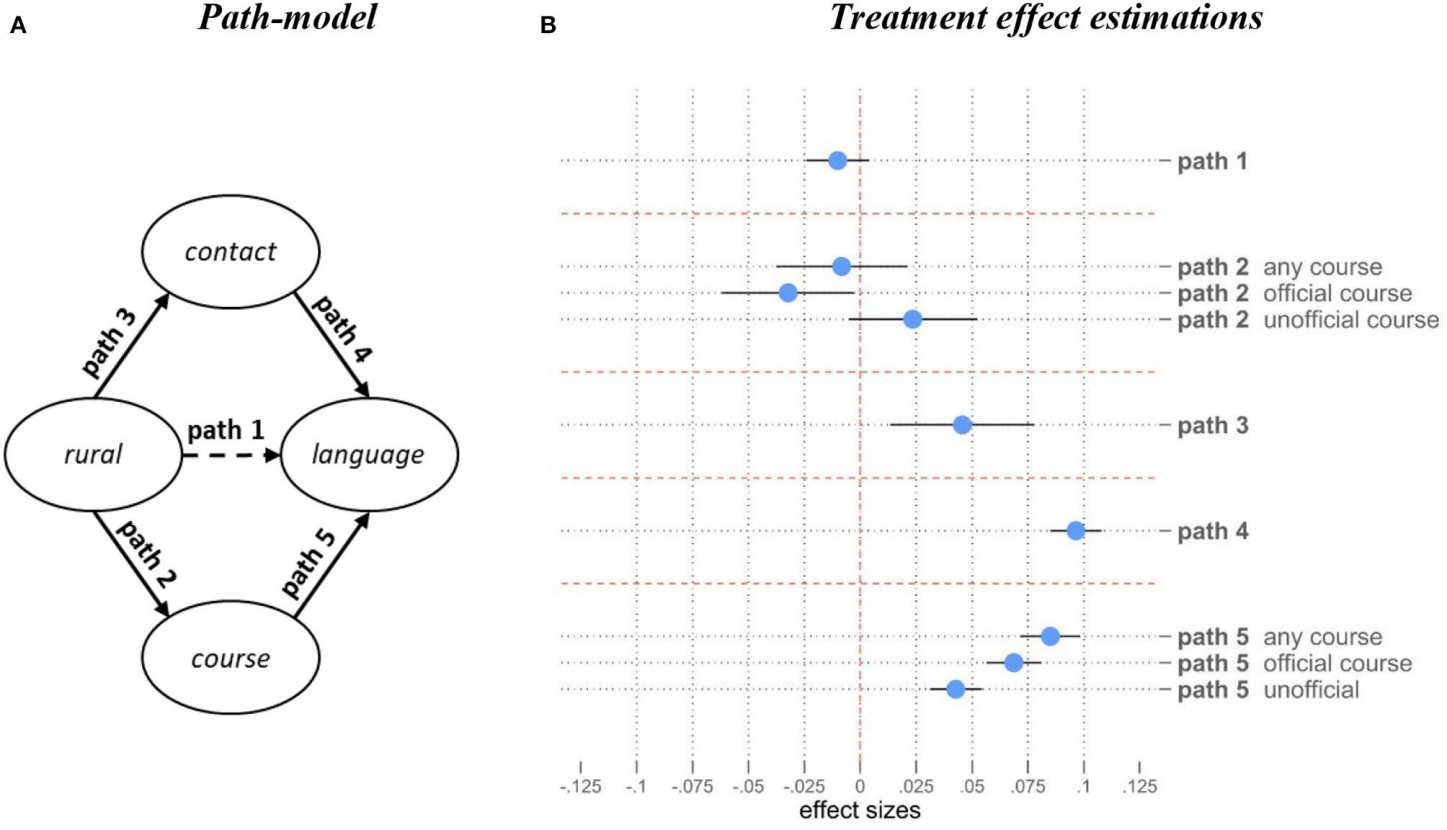
A model for rural language acquisition – Treatment Effect Estimations. **(A)** Displays the theoretical model described in detail in section Theory, **(B)** shows average treatment effect (ATE) coefficients with their 95% confidence-intervals resulting from 9 separate regressions using the regression adjustment method (including population weights). Outcomes are all scaled as binary (0, 1), language-proficiency is scaled as an index taking values between 0 and 1 (path 1, 4, 5). Non-displayed controls are included for respondents' sex, age, educational-levels, number of children, country of birth, years since immigration, legal status, partnership status and moving indicator. *N* = 13,187 observations.

Figure 3 illustrates average treatment effects of all hypothesized pathways for the causal (total) effect of rural location on refugees' language acquisition (for a coefficient table, see Supplementary Table A1). Strikingly, there is only a comparatively small negative total effect from living in rural areas on language skills, which is also not statistically significant.

For path 2 going from rural location to course participation, the picture is heterogeneous: considering all courses combined, there is a very small insignificant effect suggesting that refugees in rural areas do not have lower access to language courses compared to more urban areas. However, when we disaggregate the type of course, a different picture emerges. Living in a rural location reduces access to formal federally-organized (BAMF) courses by about 3.2 percentage points (*p* < 0.05) while course participation in other non-BAMF courses tends to be more likely in rural areas by 2.3 percentage points (ns). This may suggest that local communities offer their own language course support, perhaps partly because centrally organized courses are less accessible in rural areas.

Rural location has significantly positive effects on refugees' contact with Germans (path 3): frequent intergroup interactions (several times per week or daily) 4.6 percentage points more likely compared to refugees' living in urban areas. This may demonstrate altered opportunity structures with regards to intergroup contact across regions. Furthermore, the presence of contact has strong effects on refugees' language skills (path 4): refugees reporting more contact with Germans show a significant increase of 0.097 scale-points in language evaluation as compared to refugees who do not report it. Last, the participation in language courses has significant and strong positive effects on language skills for all forms of courses. The strongest effects are present for the combined specification of course participations with +0.085 scale points in language skills (0–1), followed by official courses (+0.069) and unofficial courses (+0.043).^3^

### Further Analyses and Robustness Checks

#### Measurements

While the main analyses distinguished between different types of courses, it is also conceivable that contact with Germans differs in frequency and effect on language acquisition depending on whether the contact takes place at work, among friends, or in the neighborhood. Thus, some forms of contact may occur relatively frequently, but the intensity of interaction and the depth of possible topics of conversation may remain relatively superficial. The refugee sample does not distinguish between contact with Germans in different spheres of life until the start of the second wave. Supplementary Figure A2 therefore presents the results for this reduced sample from wave 2 onward (8,703 cases). Specifically, the sample includes information on contact with German friends, German neighbors and German colleagues (including class mates at school/university) (SOEP Group, 2020). Regarding the effect of a rural place of living on these contact forms, there are no substantial differences visible between contact forms. All forms of contact except those with German friends are significantly positively affected. This may suggest that making friends is to some extent generally a greater hurdle than establishing other forms of contacts. When friendships with Germans could be established, however, these have a particularly strong effect on language skills and clearly outperform potentially more casual contacts such as those with neighbors. Contacts with Germans at work, school or university also have relatively strong effects on language acquisition (Supplementary Figure A2). Another factor which may affect contact quality arise from the openness toward diversity and migration within the local majority population. Therefore, based on the smallest regional units available to us, the 96 so-called “regional spatial regions,” we added the federal Bundestag election results (2017) of the right-wing populist AfD party in quartiles to the analysis and calculated the contact effects on language acquisition for each quartile separately (Supplementary Figure A3). Results show slightly weaker contact effects on language acquisition in regions with high AfD results (+0.078 scale-points) as compared to regions with low AfD-results (+0.099 scale-points) although differences are not significant.

Self-assessments of language skills are controversial with regards to their strengths in reflecting objective language skills (Edele et al., 2015). Studies that have directly compared subjective language assessments of second languages with objective language tests conclude that subjectively assessed language proficiency is relatively accurate when objective levels of proficiency (i.e., levels that would be identified by generalized tests) are either low or high (Ma and Winke, 2019). However, in the process of language learning from low to intermediate proficiency, the complexity in dealing with the language increases, while at the same time it is not yet fully apparent how far the path to very good proficiency actually still is. This can lead to misjudgments, especially in the case of intermediate skills, tending to take the form of an underestimation of one's own language skills (Edele et al., 2015; Ma and Winke, 2019). Thus, we run further robustness checks using interviewers' evaluation on respondents' German skills which are also available in the data (Supplementary Figure A4). We re-run all models from main analyses for paths in which language-skills are involved. The overall path from rurality to language acquisition is not significant in the interviewer estimate, as in the main models. Interestingly, as effects are measured by interviewer assessment, courses have smaller effects on language skills and contact with Germans has stronger effects on language skills. One interpretation of these slight deviations from main results is that language acquisition via social contacts may take place more subconsciously than that in language courses, where an explicit confrontation with the foreign language takes place. Language skills gained through social contacts may be less strongly expressed in a change in self-assessment relative to skills gained through course attendance as refugees made a deliberate effort to improve their language skills by attending a course which may lead them to overestimate their skills to reduce cognitive dissonance. Overall, this check may indicate that our analyses overestimate course effects and underestimate contact effects.

#### Varying DAG Assumptions

A path between the two moderators of intergroup contact (M1) and course visits (M2) is theoretically plausible in both directions (see Figure 1). Individuals in courses may have less time to meet Germans. Alternatively, contacts to Germans may facilitate finding and completing a language course. The DAG logic only allows one-way (“directed”) paths as each moderator would otherwise become a “collider” and threaten causal interpretation of pathways 4 and 5 (see Figure 1). For our main analyses, we blocked this path by controlling for the respective other variable when estimating effects on language acquisition (path 4 and 5). In further analyses presented in Supplementary Figure A5, we softened this restriction by not controlling for the other variable. Strikingly, there are no major differences in effect sizes observable between both model assumptions. This indicates that recruitment into courses via intergroup contacts or, reversely, fewer contacts resulting from time spent in courses – if at all – are minor pathways present in the data.

#### Risk of Reverse Causality

Another robustness check addresses issues of reverse causality. Our argument hinges on the assumption that both contacts to Germans and participation in courses have positive effects on language acquisition (paths 4 and 5). Our results confirm this assumption empirically. However, it is possible that better language skills lead to more contacts to Germans and better access to courses rather than vice versa. To assess this possibility, we run separate models (Supplementary Figure A6), taking advantage of the panel-structure of the SOEP by comparing 2-wave panels of treated individuals starting a course/contact vs. non-treated. By using two-way FE-regressions on this 2-wave data structure, we achieve a clear before-after estimation (Allison, 2009; Goodman-Bacon, 2018) mitigating the risk of reverse causality. The results illustrate that the positive effects of course attendance and contacts on German language skills are clearly visible also when explicitly modeling the temporal processes in which events occur.

#### Estimation

As a final step, we check whether effect directions are sensible toward our chosen estimation approach for obtaining ATE's. Therefore, as alternative to “regression adjustment,” we also provide estimates using two more approaches: inverse-probability-weighting (IPW) and inverse-probability-weighting regression adjustment (IPWRA). A side-by-side comparison is provided in Supplementary Figure A7. Ultimately, all methodological approaches yield very similar results, strengthening the claim that our demonstrated associations are robust to different estimation procedures.

## Conclusion

This study explored the potential disadvantage that asylum seekers and refugees may face in terms of language acquisition when being allocated to rural areas after arriving in Germany (i.e., “rural penalty”). We propose a causal model based on DAGs and established language learning models and test our hypotheses using large survey data from Germany (SOEP Group, 2020). We find that asylum seekers and refugees in rural areas do not have lower language skills compared to urban contexts (null effect). We find that asylum seekers and refugees in rural areas benefit from higher levels of interaction with German speakers while the disadvantage in terms of access to structured language courses appears minor. Overall, the results support a “compensation effect” whereas migrants compensate small disadvantages in terms of access to courses with higher exposure to Germans.

These results have implications both for academic and policy discussions. Germany received several million asylum seekers since 2012. Migrants often came from war torn countries with – on average – lower educational backgrounds. Integrating asylum seekers and refugees into society and allowing for equal participation is a major challenge. Acquiring the German language is key to integration and the largest area of public investment by the government. In this context, it is striking that the evidence on how local contexts influence integration outcomes is severely limited despite much debate regarding the issue. Rather than testing very narrow hypotheses, our approach allowed us to study how various mechanisms may offset each other within a more comprehensive causal model of language acquisition. Our findings are consistent with previous literature in the sense that we find large positive effects of both contacts to Germans and participation in language courses on language acquisition. However, we show that these mechanisms are more or less pronounced depending on the local context.

The policy debate often centers on the allocation scheme of asylum seekers in Germany (Königstein key) and the degree to which it produces winners and losers in terms of integration. We find that rural areas do not necessarily disadvantage migrants in terms of language acquisition. This finding is important as allocation of migrants to rural areas has been discussed in the context of reviving areas suffering from demographic decline. The results also suggest that policymakers can further promote language acquisition of asylum seekers and refugees by improving access to formal language courses in rural areas and by facilitating interaction with Germans in urban areas. Especially the latter informal mechanism via social contacts is often neglected in the political debate, with an overly rigid focus on language courses instead. Yet intergroup contacts can also achieve other socially desirable effects in addition to migrants' language acquisition, such as reducing xenophobic attitudes within majority groups (Savelkoul et al., 2017; Khalil and Naumann, 2021). Therefore, it could also be part of a targeted integration policy, for example, to select accommodation for refugees according to local contact opportunities and to specifically avoid too much ethnic segregation (Ziller and Spörlein, 2020).

The study faced two main limitations. First, our findings are based on observational data which limits the approach to establish causality. However, we attempted to make our causal assumptions explicit using DAGs. To further strengthen our causal claims, we benefit from the allocation policy in Germany which randomly allocates asylum seekers across regions mirroring a natural experiment. In the Supplementary Material, we also address reverse causality issues using panel fixed effects models. Second, the data only contains few years since asylum seekers arrived in Germany. Future research should study the long-term effects of being allocated to rural areas on language acquisition. In addition, we encourage further research to explore “rural penalties” with respect to other relevant integration outcomes such as employment, education, health, social exclusion and life satisfaction.

Despite these limitations, this study offers a comprehensive view of different pathways of language acquisition among asylum seekers and refugees and how they may differ between rural and more urban areas.

## Data Availability Statement

Publicly available datasets were analyzed in this study. This data can be found at: https://www.diw.de/en/diw_01.c.357906.en/soep_order_form_mod.html.

## Ethics Statement

Ethical review and approval was not required for the study on human participants in accordance with the local legislation and institutional requirements. The participants provided their written informed consent to participate in this study.

## Author Contributions

SK: concept, design, analysis, and writing. JT: concept, design, and writing. UK: design. All authors contributed to the article and approved the submitted version.

## Funding

Staff funding was provided by the German Federal Ministry for Education and Research. Funding for open access provided by the Deutsche Forschungsgemeinschaft (DFG, German Research Foundation) – Projektnummer 491466077.

## Conflict of Interest

The authors declare that the research was conducted in the absence of any commercial or financial relationships that could be construed as a potential conflict of interest.

## Publisher's Note

All claims expressed in this article are solely those of the authors and do not necessarily represent those of their affiliated organizations, or those of the publisher, the editors and the reviewers. Any product that may be evaluated in this article, or claim that may be made by its manufacturer, is not guaranteed or endorsed by the publisher.

## Footnotes

^1^In collaboration with individual German regions (Bundeslaender), researchers from the University of Hildesheim and the Friedrich-Alexander-Universität Erlangen-Nürnberg are currently developing an algorithm based mechanism to distribute refugees to districts (see www.matchin-project.de).

^2^Within the observation window between 2016 and 2019, there were 176 to 340 thousand annual course starters, making this course form by far the most frequent one in Germany (Bundesamt für Migration und Flüchtlinge 2021).

^3^One reason may be that this variable also includes relatively advanced course forms like language courses preparing participants immediately prior to labor market entry. E.g. about 8% reporting any course visit, report (among others) the “ESF-BAMF” course for occupational language training.
