# Supplementary material for: Is There a Rural Penalty in Language Acquisition? Evidence From Germany's Refugee Allocation Policy
Source: Front Sociol. 2022 Jun 2;7:841775. doi: 10.3389/fsoc.2022.841775 (PMC9201823; doi:10.3389/fsoc.2022.841775)
Supplement: Supplementary file 1 [file Data_Sheet_1.docx]

Supplementary Material

| 1. ***Path-model*** | 1. ***Treatment effect estimations*** |
| --- | --- |
| 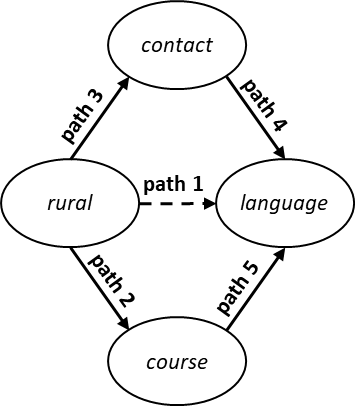 | 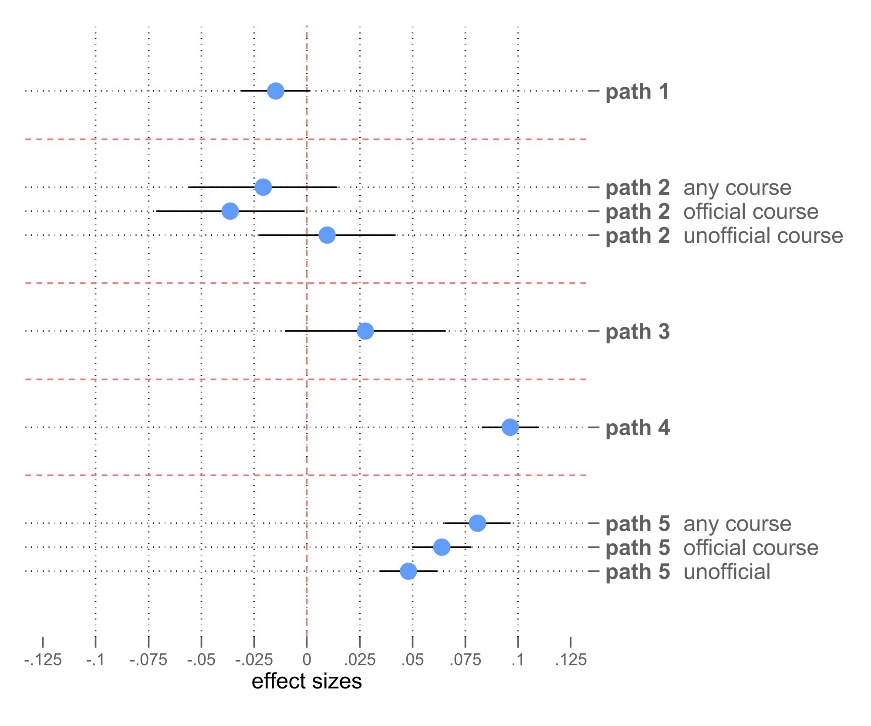 |
| **FIGURE A1 \|** Additional covariates on intention to move to rural areas.  Panel (a) displays the theoretical model described in detail in section 2, panel (b) shows average treatment effects (ATE) treatment effect coefficients with their 95-% confidence-intervals resulting from 9 separate regressions using the regression adjustment method (including population weights). Outcomes are all scaled as binary (0–1), language-proficiency is scaled as an index taking values between 0 and 1 (path 1, 4, 5). Non-displayed controls are included for respondents’ sex, age, educational-levels, number of children, country of birth, years since immigration, legal status, partnership status, moving indicator and – additionally in this consideration – three indicators on the intention to move to rural areas: having lived in a rural area or small town as opposed to (large) cities (1) prior to immigration (2) during childhood and (3) the preference to live on the countryside in general. N = 9,685 observations. | |

| 1. ***Path-model*** | 1. ***Treatment effect estimations*** |
| --- | --- |
| 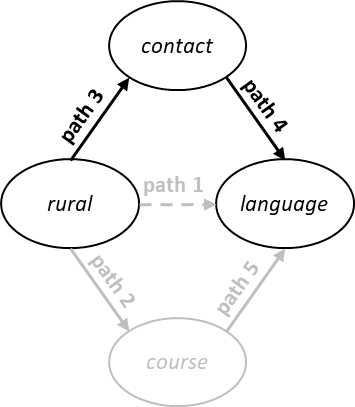 | 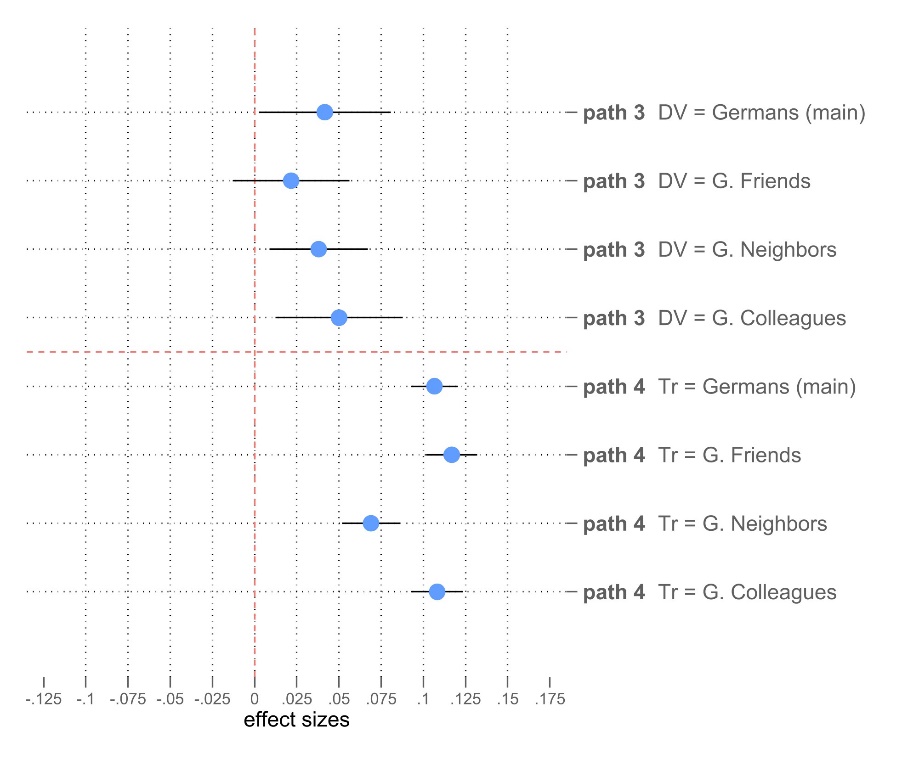 |
| **FIGURE A2 \|** Multiple forms of contact.  Panel (a) displays the theoretical model as derived in section **Error! Reference source not found.**, panel (b) shows average treatment effects (ATE) treatment effect coefficients with their 95-% confidence-intervals resulting from 8 separate regressions using the regression adjustment method (population weights). Non-displayed controls are included for respondents’ sex, age, educational-levels, number of children, country of birth, years since immigration, legal status, partnership status and moving indicator. Listwise deletion is applied across all models, resulting in one sample-size (N = 8,703). | |

| 1. ***Path-model*** | 1. ***Treatment effect estimations*** |
| --- | --- |
| 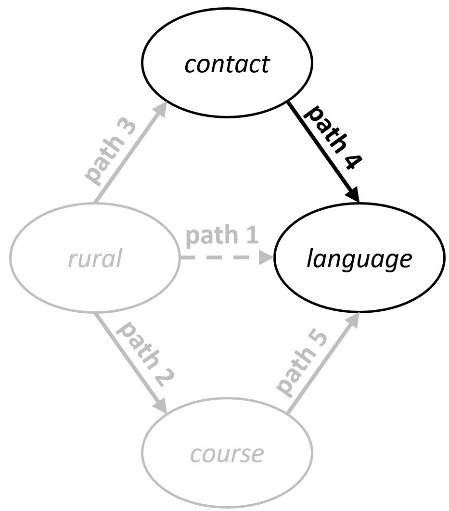 | 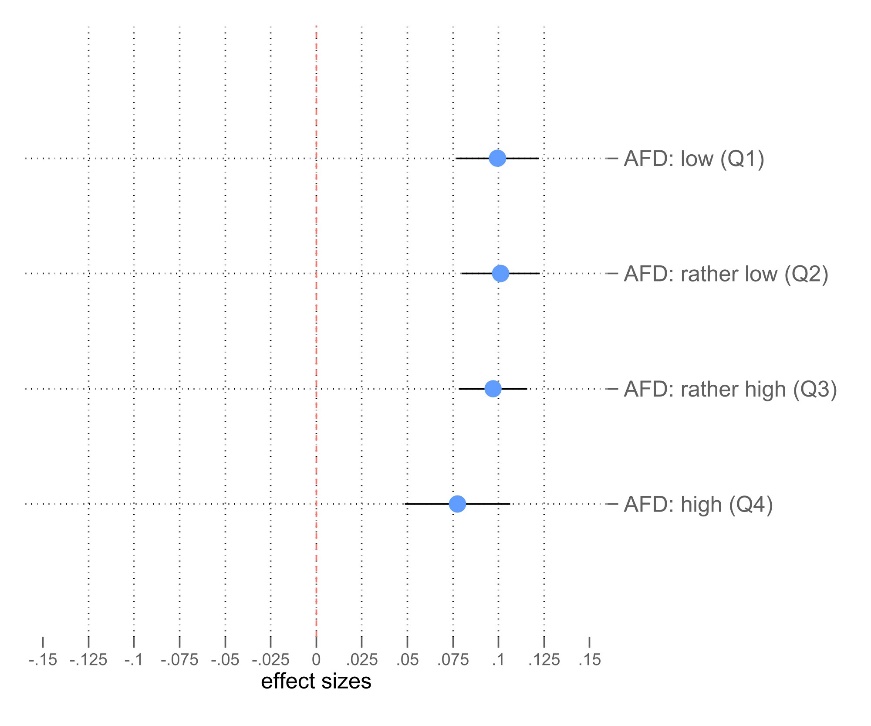 |
| **FIGURE A3 \|** Moderators of contact effects: regional hostility.  Panel (a) displays the theoretical model described in detail in section 2, panel (b) shows average treatment effects (ATE) treatment effect coefficients with their 95-% confidence-intervals resulting from 4 separate regressions, representing regional Quantiles in right-wing party’s “Alternative für Deutschland (AfD)” results in federal elections 2017 based on 96 geographic units within Germany (“Raumordnungsregionen”). Outcome language-proficiency is scaled as an index taking values between 0 and 1. Non-displayed controls are included for respondents’ sex, age, educational-levels, number of children, country of birth, years since immigration, legal status, partnership status, moving indicator. N(Q1) = 3,730; N(Q2) = 3,728; N(Q3) = 3,708; N(Q4) = 2,021. | |

| 1. ***Path-model*** | 1. ***Treatment effect estimations*** |
| --- | --- |
| 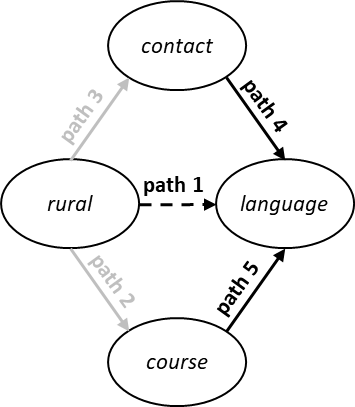 | 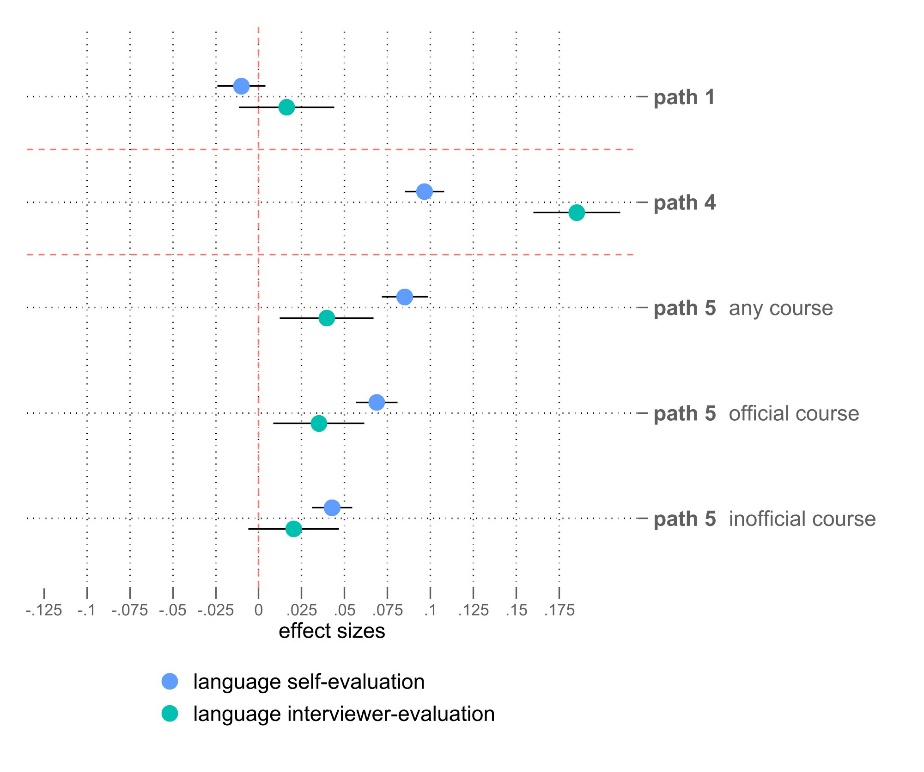 |
| **FIGURE A4** \| Language self-evaluation vs. interviewer evaluation.  Panel (a) displays the theoretical model as derived in section **Error! Reference source not found.**, panel (b) shows average treatment effects (ATE) treatment effect coefficients with their 95-% confidence-intervals resulting from 10 separate regressions using the regression adjustment method (population weights). Non-displayed controls are included for respondents’ sex, age, educational-levels, number of children, country of birth, years since immigration, legal status, partnership status and moving indicator. Listwise deletion is applied across all models, resulting in one sample-size (N = 13,187). | |

| 1. ***Path-model*** | 1. ***Treatment effect estimations*** |
| --- | --- |
| 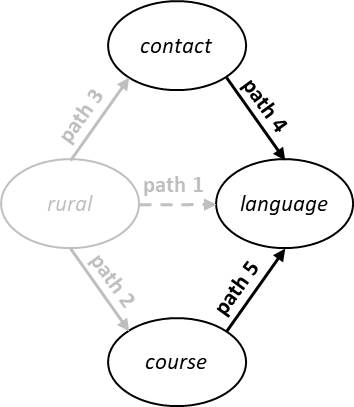 | 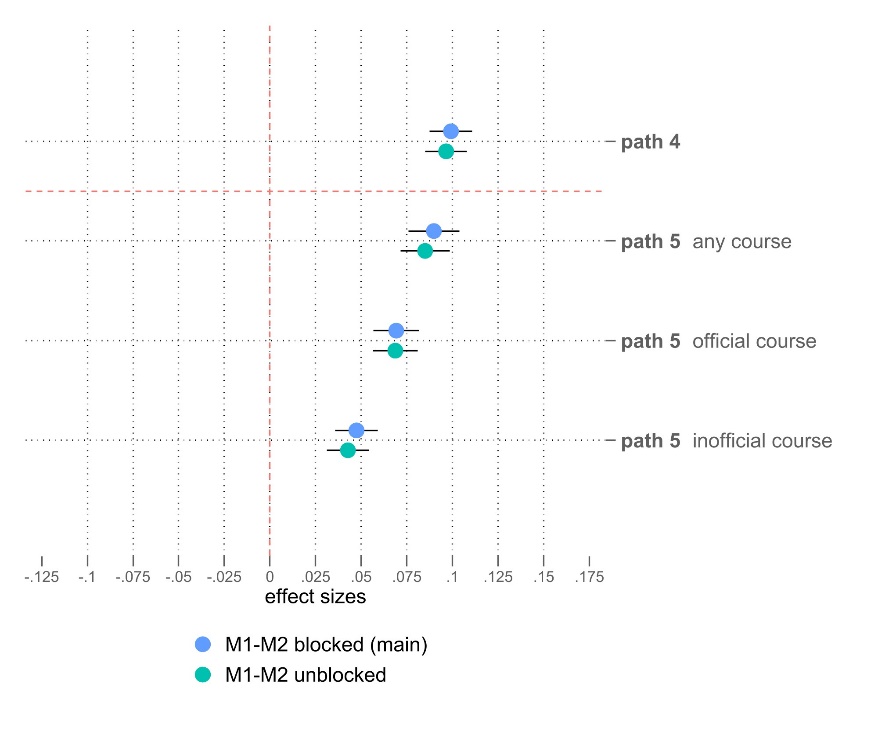 |
| **FIGURE A5** \| Varying DAG assumptions.  Panel (a) displays the theoretical model as derived in section **Error! Reference source not found.**, panel (b) shows average treatment effects (ATE) treatment effect coefficients with their 95-% confidence-intervals resulting from 8 separate regressions using the regression adjustment method (population weights). Non-displayed controls are included for respondents’ sex, age, educational-levels, number of children, country of birth, years since immigration, legal status, partnership status and moving indicator. Listwise deletion is applied across all models, resulting in one sample-size (N = 13,187). | |

| 1. ***Path-model*** | 1. ***Treatment effect estimations*** |
| --- | --- |
| 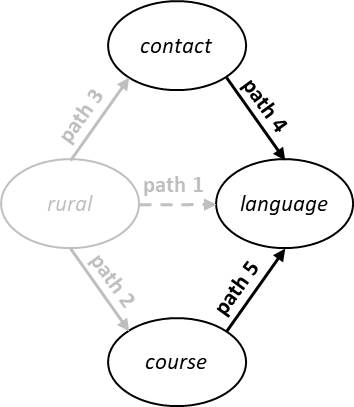 | 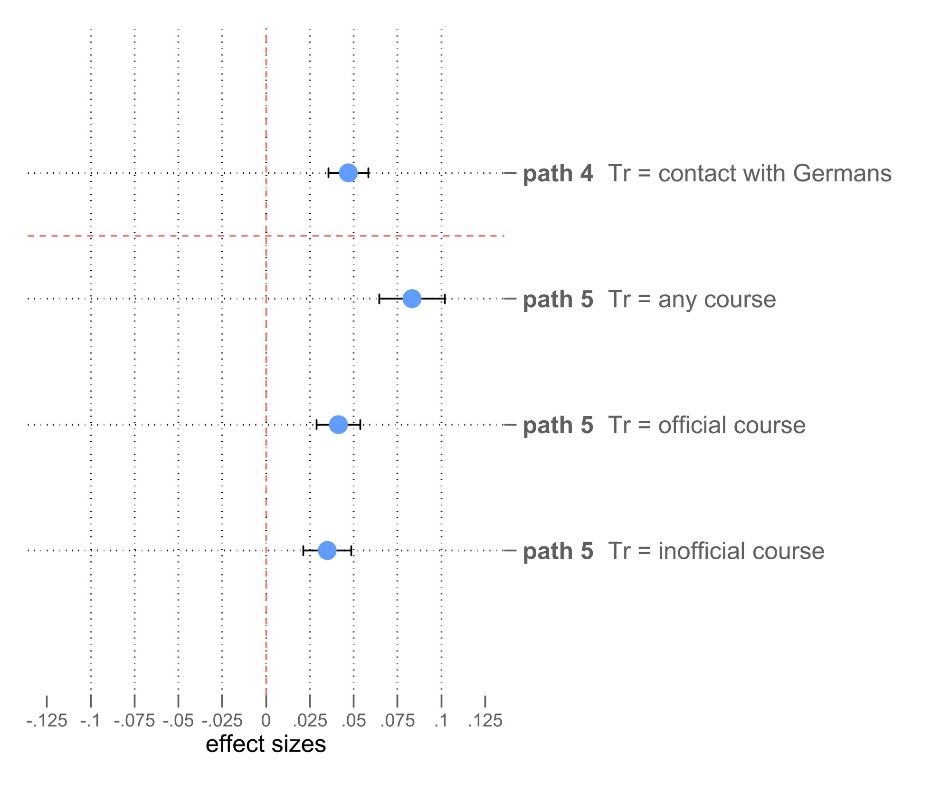 |
| **FIGURE A6** \| DiD modelling of path 4 and 5 with multiple treatment timing.  Panel (a) displays the theoretical model as derived in section **Error! Reference source not found.**, panel (b) shows results following four separate two-way fixed regressions. Analyses cases are considered if they represent one year prior or one year since reported treatments (treatment group) or if individuals do not report any treatment (control group). Due to different frequencies of treatments, samples vary in size likewise (N = 3,106 – 6,894). Time-varying controls are included: years since immigration, partnership indicator, moving indicator, legal status and employment status. | |

| 1. ***Path-model*** | 1. ***Treatment effect estimations*** |
| --- | --- |
| 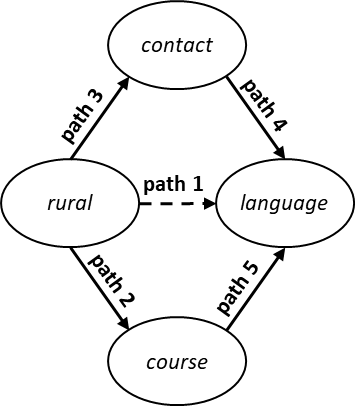 | 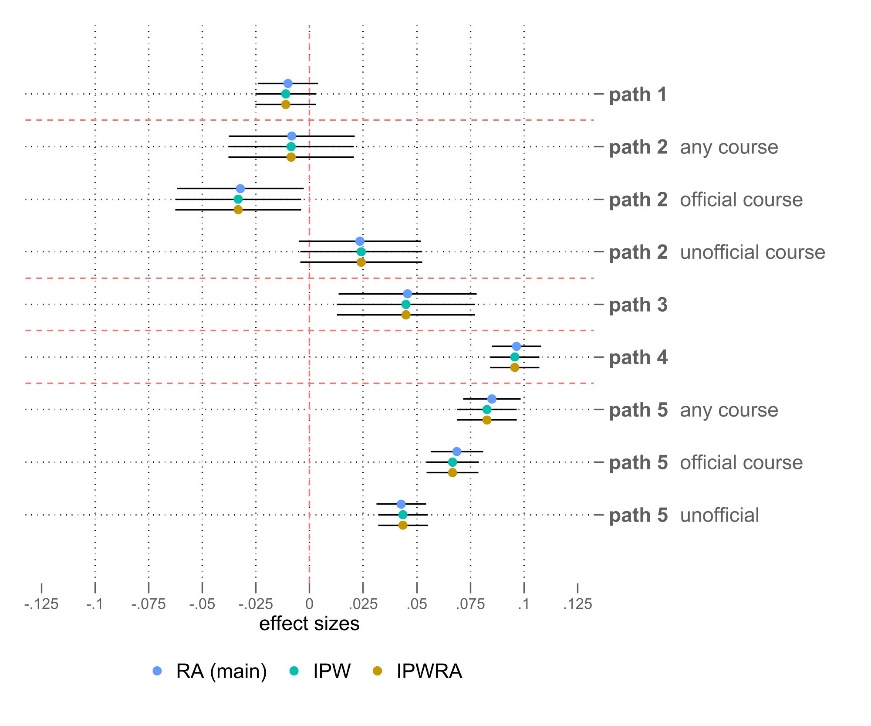 |
| **FIGURE A7 \|** Varying estimation methods.  Panel (a) displays the theoretical model described in detail in section 2, panel (b) shows average treatment effects (ATE) treatment effect coefficients with their 95-% confidence-intervals resulting from 27 separate regressions using the regression adjustment method (including population weights). Outcomes are all scaled on 0–1, language-proficiency-index can take intermediate values (path 1, 4, 5), contact with Germans and course visits are dichotomized. Colors indicate different methods for estimating treatment effect: regression adjustment (RA), inverse-probability-weighting (IPW) & inverse-probability-weighting-regression-adjustment (IPWRA). Non-displayed controls are included for respondents’ sex, age, educational-levels, number of children, country of birth, years since immigration, legal status, partnership status and moving indicator. N = 13,187 observations. | |

| 1. ***Path-model*** | 1. ***Treatment effect estimations*** |
| --- | --- |
| 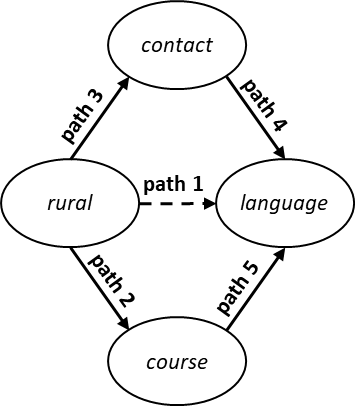 | 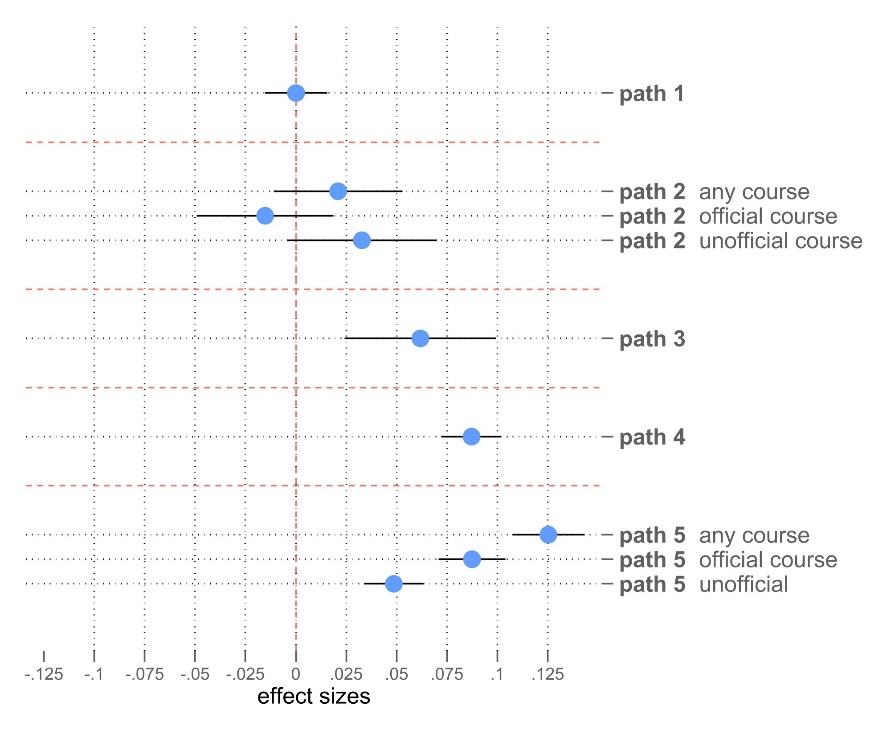 |
| **FIGURE A8 \|** Use only respondents’ first wave.  Panel (a) displays the theoretical model described in detail in section 2, panel (b) shows average treatment effects (ATE) treatment effect coefficients with their 95-% confidence-intervals resulting from 9 separate regressions using the regression adjustment method (including population weights). Outcomes are all scaled as binary (0–1), language-proficiency is scaled as an index taking values between 0 and 1 (path 1, 4, 5). Non-displayed controls are included for respondents’ sex, age, educational-levels, number of children, country of birth, years since immigration, legal status, partnership status and moving indicator. N = 6,985 observations. | |

| **MODEL** | **PATH** | **COEF** | **CI_LB** | **CI_UB** |
| --- | --- | --- | --- | --- |
| 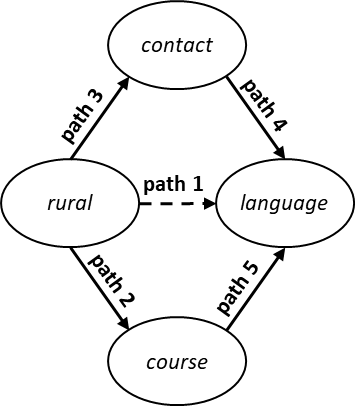 | path 1 | -0.010 | -0.024 | 0.004 |
|  | path 2: any course | -0.008 | -0.038 | 0.021 |
|  | path 2: official course | -0.032 | -0.062 | -0.003 |
|  | path 2: unofficial | 0.023 | -0.005 | 0.052 |
|  | path 3 | 0.046 | 0.014 | 0.078 |
|  | path 4 | 0.097 | 0.085 | 0.108 |
|  | path 5: any course | 0.085 | 0.072 | 0.098 |
|  | path 5: official course | 0.069 | 0.056 | 0.081 |
|  | path 5: unofficial | 0.043 | 0.031 | 0.054 |
| **TABLE A1** \| A model for rural language acquisition – Treatment Effect Estimations  Presented are the average treatment effects (ATE) coefficients with their 95-% confidence-intervals resulting from 9 separate regressions using the regression adjustment method (including population weights). Outcomes are all scaled as binary (0–1), language-proficiency is scaled as an index taking values between 0 and 1 (path 1, 4, 5). Non-displayed controls are included for respondents’ sex, age, educational-levels, number of children, country of birth, years since immigration, legal status, partnership status and moving indicator. N = 13,187 observations. | | | | |
